# Impact of Cisplatin Dosing Regimens on Mammary Tumor Growth in an Animal Model

**DOI:** 10.33696/cancerbiology.1.004

**Published:** 2020-06-15

**Authors:** James A. Koziol, Theresa J. Falls, Jan E. Schnitzer

**Affiliations:** Proteogenomics Research Institute for Systems Medicine (PRISM), La Jolla, California 92037, USA

**Keywords:** Tumor growth, Cisplatin chemotherapy, Mathematical models, Metronomic drug delivery

## Abstract

**Background:**

We have recently introduced a modification of the seminal Simeoni model for tumor growth, the modification entailing the incorporation of delay differential equations into its formulation. We found that the modification was competitive with the Simeoni construct in modeling mammary tumor growth under cisplatin treatment in an animal model.

**Methods:**

In our original study, we had two cohorts of animals: untreated, and treatment with bolus injection of cisplatin on day 0. We here explore how modifications in the cisplatin dosing scheme affect tumor growth in our model.

**Results:**

We found that modest fractionation dosing schemes have little ultimate impact on tumor growth. In contrast, metronomic dosing schemes seem quite efficacious, and might yield effective control over tumor progression.

**Conclusions:**

With regard to cisplatin as single agent chemotherapy, a minimum level of drug for a prolonged period of time seems more critical than rapid achievement of a very high dose for a shorter time frame for deterring tumor growth or progression. Exploration of tumor dose schedules with mathematical models can provide valuable insights into potentially effective therapeutic regimens.

## Introduction

In a recent paper [1], we introduced a variant of the classical Simeoni tumor growth model [2–4], and illustrated its value in assessing tumor growth in a reproducible mouse model for mammary tumors. Our modification consisted of incorporating delay differential equations in the mathematical formulation of the Simeoni model, to represent the delay in drug action often observed under chemotherapeutic or immunotherapeutic regimens. Simeoni and colleagues had postulated that a drug’s action is not instantaneous but delayed, as tumor cells pass through progressive stages of damage before ultimate elimination. Simeoni modeled this delay in drug action by means of a series of delay compartments through which the damaged cells transit before elimination. In contrast, we incorporated a delay differential equation to represent the series of delay compartments, and found that our formulation was competitive with Simeoni in our experimental mouse model for mammary tumors.

In this note, we return to our mouse model, and investigate how modifications of dosing regimens affect tumor growth. We briefly review methodology in the following section, and then compare various modifications of our original chemotherapeutic regimen with cisplatin relative to tumor growth with our variant of the Simeoni model. We conclude that dosing schemes are quite relevant to achieving optimal tumor regression, with mathematical models such as Simeoni’s affording valuable insights toward realizing this goal.

## Methods

We begin with the *in vivo* tumor growth experiments described in detail in [1]. We had utilized the FVB/NTg(MMTVNeu)202Mul/J mouse model of mammary tumor growth, and reported on a cohort of 40 female mice, 21 controls (no treatment), and 19 mice given a single dose of cisplatin (5 mg/kg) on day 0. Tumor volumes were then recorded daily (excepting weekends, holidays) over the course of the experiment.

Since the focus of this note is not model development, we refer the interested reader to our previous publication [1] for explication of the Simeoni model and our variant. Therein we compared the Simeoni model and our variant relative to assessing the primary experimental outcome, tumor volumes, and found that our variant was quite competitive with the Simeoni model in this regard. Our model fits were implemented in Monolix (Lixoft, Antony, France), a suite of programs for model-based drug development entailing population analysis, pharmacometrics, pre-clinical and clinical trial modeling, and simulation. Here, Monolix provided estimation of the population parameters in our models, by means of nonlinear mixed effects algorithms. In this note, we use another program in the Monolix suite, namely, Mlxplore, for exploration and visualization of our pharmacokinetic model with the fitted population parameters from Monolix. That is, we utilized Mlxplore to simulate our model under different dosing regimens in comparison to the original single bolus dose of cisplatin on day 0. We report on these simulations in the following section.

## Results

**Figure 1:**
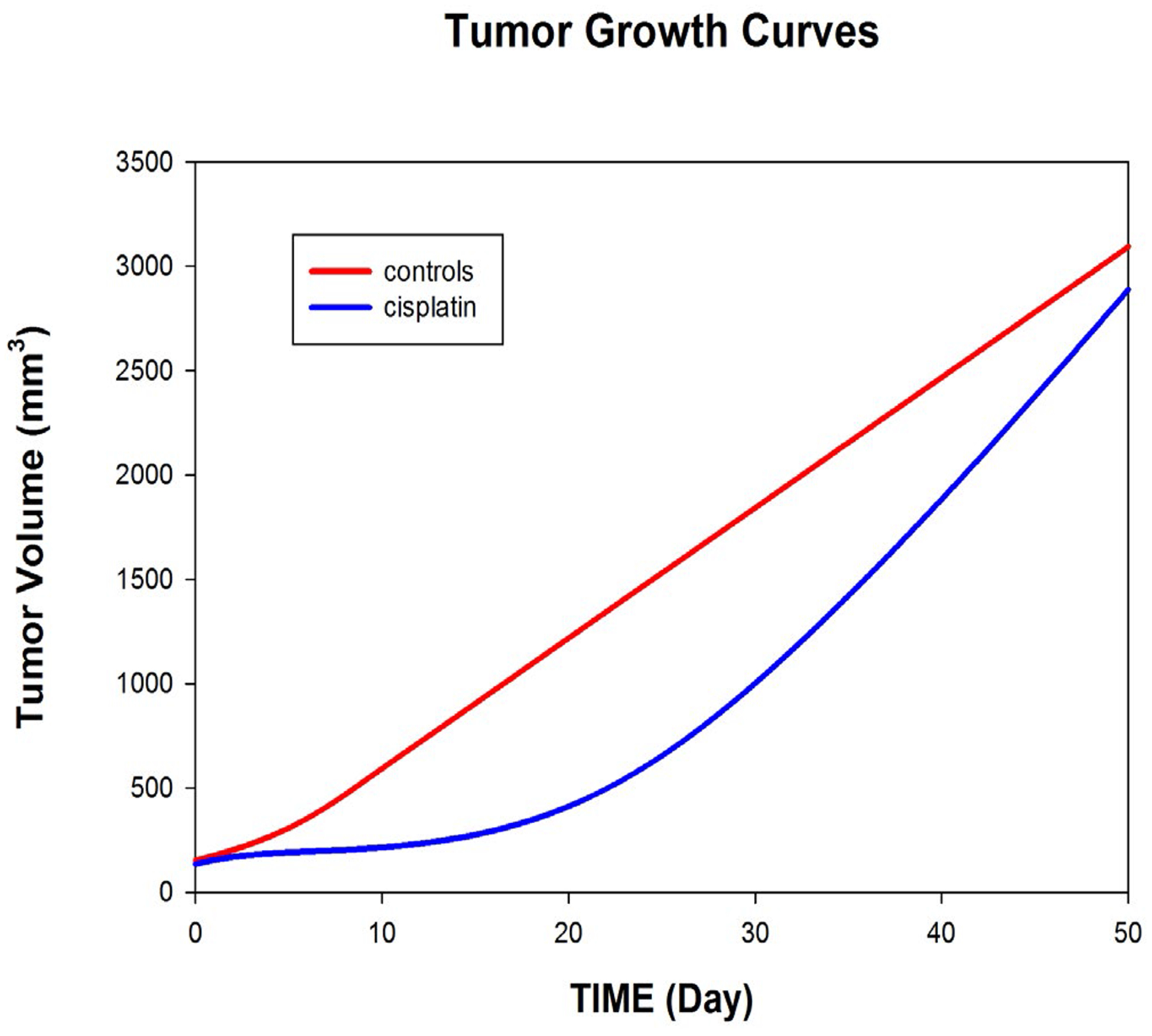
Population fits to tumor growth over time among 21 control animals (untreated) and 19 animals receiving a bolus injection of cisplatin (5 mg/kg) on Day 0.

We first summarize the population fits to tumor growth for the controls (untreated) and the cisplatin group (5 mg/kg, day 0). As shown in Figure 1, bolus injection of cisplatin on day 0 retards tumor growth initially, but tumor growth then returns to levels comparable to the control group.

We next undertook a simulation study, to investigate five different dose schedules for cisplatin: 5 mg/kg cisplatin on day 0; 2.5 mg/kg on day 0 and day 7; 1.66 mg/kg on day 0, day 7, and day 14; 1.66 mg/kg on day 0, day 10, day 20; 1.66 mg/kg on day 0, day 14, day 28; 1.66 mg/kg on day 0:7:42. These dose schedules were chosen so as to satisfy a total cisplatin dose of 5 mg/kg as with the original treatment group.

Depending on one’s time horizon, different conclusions might be drawn concerning relative efficacies of the various dosing schedules. If one adopts an early horizon, 20 days or less, then bolus injection on day 0 would likely be judged optimal, and the dosing regimen spread out to days 0, 14, and 28 would likely be considered inferior to the other regimens. In contrast, the relative orderings of the regimens would be inverted as one proceeds from day 20 to day 50. None of the regimens achieves effective control over tumor growth at day 50.

We also investigated two multiple dosing schemes: the first comprised a metronomic dose schedule of 0.714 mg/kg cisplatin on day 0:7:42, and the second comprised a dose schedule of 1.428 mg/kg cisplatin on day 0:7:42. The first scheme entails a total cisplatin dose of 5 mg/kg, and the second entails a total cisplatin dose of 10 mg/kg. The corresponding tumor growth curves are depicted in Figure 3.

**Figure 2:**
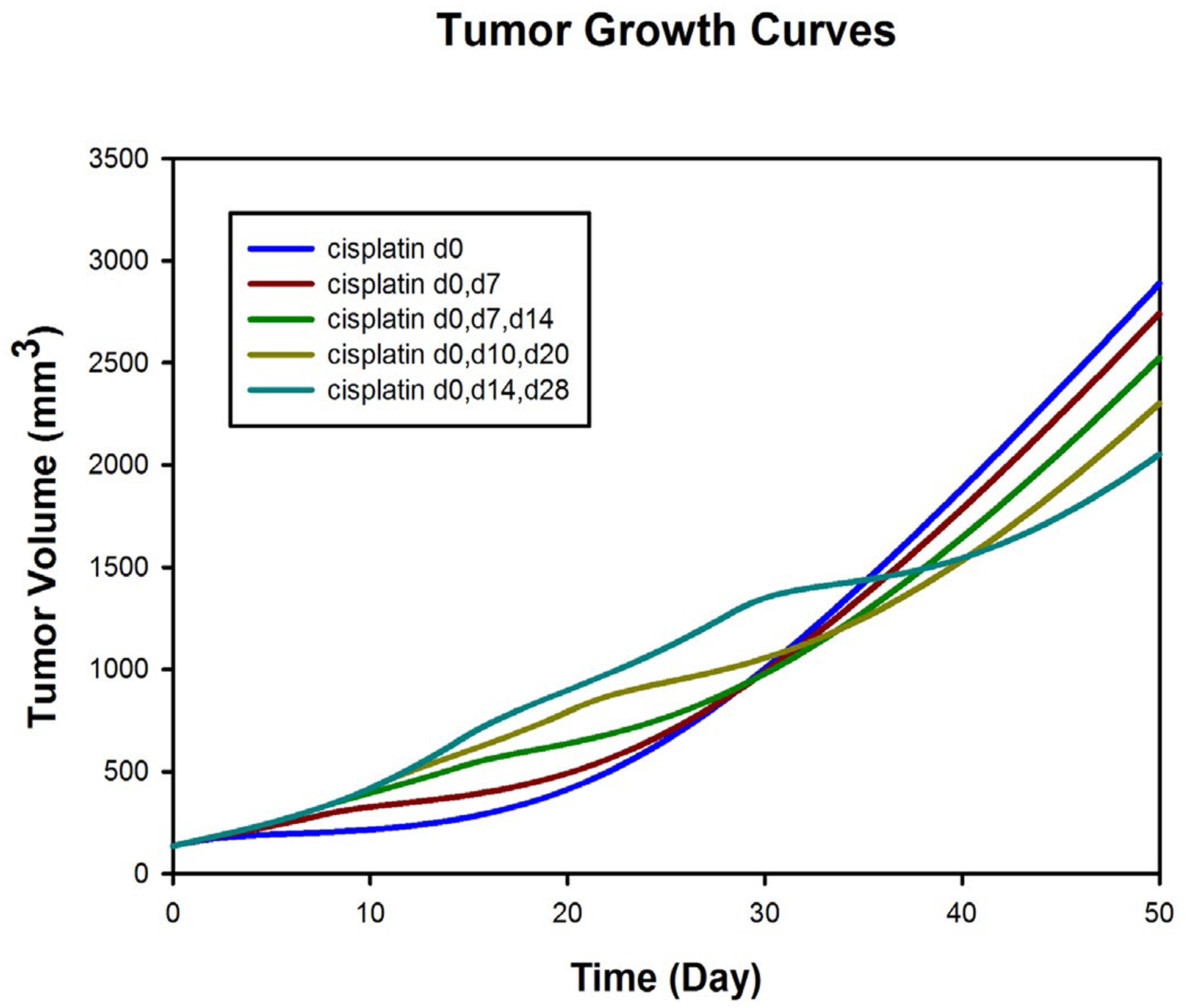
Population fits to tumor growth over time among 19 animals receiving cisplatin (total of 5 mg/kg) with different dose schedules: (i) 5 mg/kg day 0; (ii) 2.5 mg/kg on day 0 and day 7; (iii) 1.66 mg/kg on day 0, day 7, and day 14; (iv) 1.66 mg/kg on day 0, day 10, and day 20; (v) 1.66 mg/kg on day 0, day 14, and day 28.

As with Figure 2, conclusions concerning efficacious dosing schedules depend on one’s time horizon. Early (20 days) indications of the primacy of bolus injection on day 0 vanish by day 50; and, remarkably, both metronomic dose schedules achieve some degree of control over tumor growth at day 50, relative to bolus injection at day 0 or any of the periodic schemes shown in Figure 2. However, with cessation of cisplatin, one might expect the tumor growth curves to resume their upward course.

Lastly, we calculated relative areas under the cisplatin plasma concentration-time curve (AUCs) for all treatment regimens, maintaining the assumption of first order kinetics for cisplatin elimination. In this regard, we utilized the principle of superposition, whereby the total concentration of cisplatin at any time point is the sum of the remaining concentrations of each administered dose at that time. These tabulated AUC values are given in Table 1.

## Discussion

It is not at all surprising that cisplatin retards tumor growth in our animal model. Cisplatin is an alkylating agent that is cell cycle nonspecific, and has long been used in the management of breast cancer [15]. Relative to a single bolus at time 0, multidosing regimens show some advantage (Figure 2). One might infer that, at least in the case of cisplatin as single agent, a minimum level of drug for a prolonged period of time seems more critical than rapid achievement of a very high dose for a shorter time frame.

**Figure 3:**
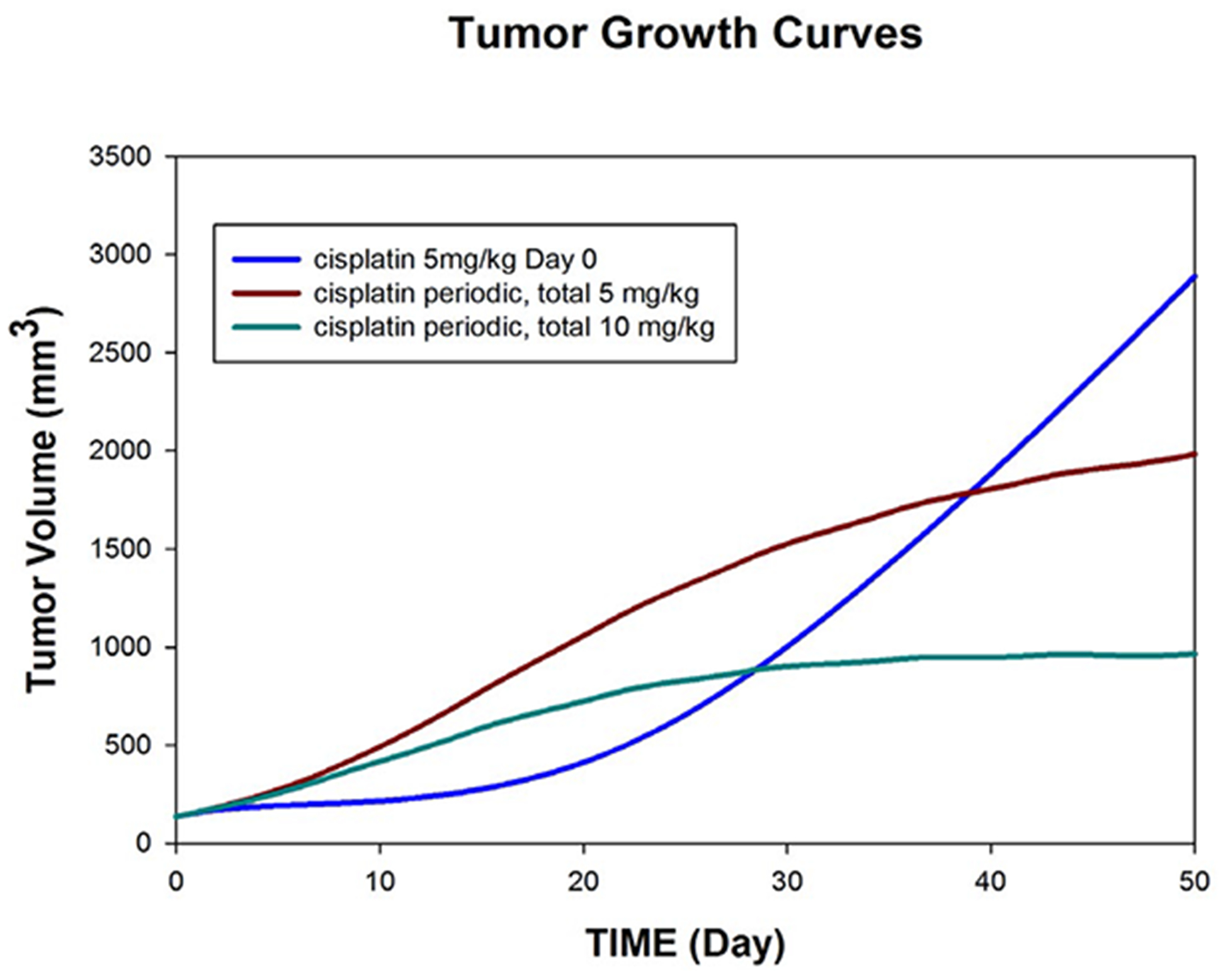
Population fits to tumor growth over time among 19 animals receiving cisplatin with different dose schedules: (i) 5 mg/kg day 0; (ii) 0.71 mg/kg on days 0, 7, 14, 21, 28, 35, and 42 (total of 5 mg/kg per animal); (iii) 1.42 mg/kg on days 0, 7, 14, 21, 28, 35, 42 (total of 10 mg/kg per animal).

Note, however, that a metronomic dosing scheme (Figure 3) shows definite improvement in retarding tumor growth relative to the other regimens. Metronomic chemotherapy [5–6] is a promising alternative to the maximum tolerated dose (MTD) paradigm which has driven many high dose chemotherapy protocols since the seminal work of Skipper [7–9]. Notably, metronomic chemotherapy appears to enjoy substantial anti-angiogenic and immunostimulatory properties compared to MTD dosing schemes [10]. As for the timing of our metronomic regimens, we note that the estimated half-life of cisplatin in the original treatment group was 6.9 days, which we took as 7 for these simulations. Hence these dose schedules were meant to achieve an eventual steady-state concentration of cisplatin, had we continued with cisplatin treatment beyond 42 days. Although our study bears out the relative benefit of a metronomic dosing scheme, we recognize that our underlying model is minimal, in that it does not take into account possible tumor heterogeneity, drug resistance, cumulative toxicity, or saturation kinetics. We refer the interested reader to [11–13] for review and development of more sophisticated mathematical models for metronomic chemotherapy.

Areas under the curve do not seem to be altogether helpful in determining an optimal dosing scheme. This should not be surprising: for example, an early study [14] found along-term intermediate dosing regimen to be as efficacious as a short-term high dose regimen for inducing remissions in children with relapsed acute lymphoblastic leukemia, even though there was about a 20-fold smaller area under the concentration-time curve with the intermediate dosing regimen compared to the high dose regimen. In our study, areas under the curve have little correspondence with tumor growth. For a fixed dose of cisplatin, maximum AUC is obtained with a single bolus at time 0, but greater impact on ultimate tumor growth is found with the other dosing regimens. Hence it is not obvious that maximizing AUC is a necessary or desirable goal of a chemotherapeutic regimen.

## Figures and Tables

**Table 1:** Relative areas under the cisplatin concentration-time curve for various dosing schedules.

Dose Schedule	AUC
Day 0	2.433
Day 0, Day 7	2.425
Day 0, Day 7, Day 14	2.411
Day 0, Day 10, Day 20	2.389
Day 0, Day 14, Day 28	2.332
Days 0:7:42	2.140
Days 0:7:42, high dose	2.280

**Note:**Areas under the concentration-time curve were calculated from day 0 to day 50. The first six dose schedules are depicted in Figures 2 and 3, and correspond to 5 mg/kg cisplatin. The last dose schedule is depicted in Figure 3, and corresponds to 10 mg/kg cisplatin.
